# The Journal of Nervous and Mental Disease

**Published:** 1879-02

**Authors:** 


### Article XVII.

### The Journal of Nervous and Mental Disease.

Our only journalistic collaborator in the field of regular medicine in this city, the *Journal of Nervous and Mental Disease*, commences with the present year its sixth volume. Though started under difficulties that might have deterred many from such an enterprise, it has risen to be the recognized organ of the neurologists of this country, and has achieved an international reputation. That is not a narrow specialty it covers, but one possessing interest for every physician who is above mere routine practice.

We take the highest pleasure in again commending our journalistic contemporary to our readers and correspondents.

---

INFLUENCE OF MOTION ON BACTERIA. B. A. Horrath. (*Pflüger's Archiv. Bd. XVII, p.* 125.)

From the fact, that bacteria injected into the circulation disappear rapidly from the blood-vessels, and are only found in the lymphatics, H. supposed, that a relative rest is necessary to the growth and multiplication of these organisms. In order to test this view, he introduced bacteria into a nutrient fluid composed of 10.0 grams neutral tartrate of ammonium, 5.0 acid phosphate of potassium, 5.0 sulphate of magnesium and 0.5 chloride of potassium in 1 litre water, (Pasteur's fluid.) Glass tubes were filled partly with this fluid, partly with air and sealed. The tubes, which were left undisturbed at a temperature of 24° C, were soon turbid with living bacteria. A number of tubes, however, were attached to a board, which was shaken about a hundred times a minute by a water motor. By this incessant motion, further growth of bacteria was checked, but if the tubes were set aside after 24 hours shaking, bacterial development again occurred. When, however, the motion was kept up for 48 hours the organisms were killed absolutely. (*Centralbl, f. Chir.* 1878, No. 43.)

WHICH ELECTRIC CURRENT TO USE IN NEURALGIA.) *Med. and Sur. Reporter.*)

Dr. Rockwell says: "I find the effects of pressure are exceedingly useful. I would not lay it down as a law, but it will be found in the great majority of cases of neuralgia that where firm pressure over the affected nerves *aggravates* the pain, the galvanic current is indicated; while the Faradic current has the greater power to relieve when such pressure does not cause an increase of pain."

